# Online Customer Experience Leads to Loyalty *via* Customer Engagement: Moderating Role of Value Co-creation

**DOI:** 10.3389/fpsyg.2022.897851

**Published:** 2022-07-28

**Authors:** Farooq Ahmad, Khurram Mustafa, Syed Ali Raza Hamid, Kausar Fiaz Khawaja, Shagufta Zada, Saqib Jamil, Muhammad Nawaz Qaisar, Alejandro Vega-Muñoz, Nicolás Contreras-Barraza, Naveed Anwer

**Affiliations:** ^1^Faculty of Management Sciences, University of Okara, Okara, Pakistan; ^2^Fatima Jinnah Women University, Rawalpindi, Pakistan; ^3^Hamdard Institute of Management Sciences, Hamdard University, Islamabad, Pakistan; ^4^Faculty of Management Sciences, International Islamic University, Islamabad, Pakistan; ^5^Business School, Henan University, Kaifeng, China; ^6^Department of Business Administration, ILMA University, Karachi, Pakistan; ^7^Faculty of Management Sciences, National University of Modern Languages, Islamabad, Pakistan; ^8^National Accountability Bureau, Peshawar, Pakistan; ^9^Public Policy Observatory, Universidad Autónoma de Chile, Santiago, Chile; ^10^Facultad de Economía y Negocios, Universidad Andres Bello, Santiago, Chile; ^11^Faculty of Management Sciences, Shaheed Zulfikar Ali Bhutto Institute of Science and Technology, Larkana, Pakistan; ^12^Lahore Business School, University of Lahore, Lahore, Pakistan

**Keywords:** online customer experience, customer loyalty, value co-creation, multi-attribute utility theory, attitude-behavior-context theory, customer engagement

## Abstract

With the increasing growth of online shopping, businesses are intertwining to establish new shopping antecedents. Customer experience has steadily become the most important source of retailers’ long-term competitive advantage *via* difference. To preserve long-term and sustained consumer loyalty, retailers must continually improve the customer experiences. This study presents a framework for online retailing in a digital environment called the Online Customer Experience-Engagement Context model in the presence of value co-creation (VCC). Data was gathered from 189 people who purchased products online. For additional analysis, testing the hypothesis, and model construction, SPSS 26 and Smart-PLS were used. The data was then evaluated further using descriptive statistics, path analysis, measurement, and structural model. The findings show that the online customer experience (measured as shopping environment, shopping procedure, staff service experience, and product experience) substantially influenced customer engagement, which improved customer loyalty. The VCC strengthened the link between online customer experience and customer engagement. It suggests that including consumers in co-creating a delightful online customer experience from time to time may be a valuable strategy for online retailers to increase customer engagement and loyalty. To create an overarching outcome, information integration theory (IIT), multi-attribute utility theory (MAUT), and the attitude-behavior-context theory (ABC) theories are converged to explain the proposed model in the study.

## Introduction

The presence of customers for online shopping is on the surge nowadays. The core factors for this trend are international dynamics, sophisticated infrastructure, the evolving lifestyle of consumers, and the mushroom growth of information communication and technology. Altogether these factors have enhanced consumers’ needs regarding updated trends and improved living compatible with socially accepted standards. The most beneficial aspect of online shopping is that it induces customer experience due to comparing products and services (Sivanesan, 2017). The customers of various age groups, particularly the youth segment of society, have exhibited a profound presence on different online sources for shopping purposes. Thus, customers’ online presence for shopping purposes has increased their shopping options enormously and widened their choice (Ellison et al., 2020).

The pervasive engagement of people in online shopping sites is attributed to ease, timeliness, and loyalty factors surfaced due to behavioral learning. While online customer experience evolves based on heterogeneous factors, it has become a kind of query for researchers and managers to examine the customer engagement process in online shopping (Gao et al., 2021). Probing into the engagement process and assessing online customer activities related to shopping may further help reduce consumers’ security concerns regarding online shopping; augment customer loyalty, brand proliferation, and general customer satisfaction. Previous research affirms that customer experience, by and large, may be attributed to various factors; however, attitudinal and behavioral engagements have underlying connections with consumers’ utilitarian and hedonic values (Barari et al., 2020). This study conjectures that while customers’ online shopping behavior is not predictable due to the technology evolution, disruption, and diffusion, what factors could moderate the relationship between customers’ engagement behavior and their online shopping experience. Furthermore, under the presently prevailing COVID phenomenon, an unequivocal surge in online economic activities has slightly strengthened consumers’ behavioral intention, decision-making for online transactions, and perception. However, this study will indeed provide a pathway to assess value co-creation (VCC) concerning online consumers’ platforms (Watson and Popescu, 2021). This will enable the managers of online ventures to harness the experience of customers in terms of valued services that may be co-created (providers and customers) on the one hand; increase the likelihood of repetitive online shopping experiences, particularly regarding purchasing habits and behavioral choices contributing to the vitality of brand on the other (Kumar and Anjaly, 2017; Rydell and Kucera, 2021).

Another essential concept of VCC is integrated as part and parcel of the theoretical framework. The concept evolved in recent times as an overarching phenomenon that refers to mutual and shared ownership of services with participatory and engagement approaches and has a predisposition for augmented customer experience in principle. VCC refers to a phenomenon that implies an active customer’s involvement psychologically and physically in crafting, utilizing, and evaluating the services. Particularly framing VCC as an integral part of online shopping activities reinforces the online shopping experience’s compass. As customers experience business context and propose value based on their perceived intentions, VCC has proved to be an interactive business concept that further outlines their attitudinal predisposition for online shopping.

The study proposes information integration theory (IIT) to examine the theoretical framework of the online shopping experience and customers’ behavior. According to the theoretical framework’s antecedents of customer experience, customer experience comprises the shopping environment, staff service environment, shopping procedure, and product experience. Customers process these antecedents as a source of information integration to encapsulate customer experience. The IIT represents customers’ attitudinal ability to assess information in the given context to have behavioral outcomes. In light of IIT, the study is most likely to see the continuity of customer attitudes developed concerning the antecedents of customer experience and their relationship with behavioral intention and engagement. The primary question in the study is how VCC helps customers devise their attitudinal predisposition to get engaged in online shopping behavior (Meilhan, 2019). In this study, the VCC construct remains to be studied as an instrumental one in light of the proposed theory. Since VCC is an arrangement whereby interactive participation and dialogue further crystalize the information, thus helping participants or shoppers to involve meaningfully in the process on a hedonic basis. Moreover, the study seeks to determine the imperatives of online customer engagement behavior that lead to consumers’ loyalty factors (Miricǎ, 2019). Hence, there is a gap existed in literature about the moderation of VCC over the relationship of online customer experience and customer engagement. Therefore, the following questions will be addressed in this study: (1) what is role of antecedents of online customer experience on the customer attitudinal and behavioral engagement?, (2) how customer attitudinal and behavioral engagement impacts the customer loyalty?, (3) what degree of effect of VCC on the relationship of antecedents of online customer experience and customer attitudinal and behavioral engagement?, and (4) how customer attitudinal and behavioral engagement mediates the relationship between antecedents of online customer experience and customer loyalty?

Conversely, the deficit on account of VCC operationalization in the given model may be endorsed by underpinning IIT. In light of the stated theories, hypothesis testing will be accomplished conveniently. Furthermore, every variable of the underlying model will have a clear underpinning relationship.

## Theory and Hypotheses Development

The nature of the products plays an integral part in the evolution and enhancement of online shopping. As a result, merchants must understand the elements that influence customers’ attitudes and the consequences, such as purchase intentions and loyalty to online stores. This study used the multi-attribute utility theory (MAUT) and the attitude-behavior-context theory (ABC) theories to investigate this. MAUT aids the decision-making process. It is used in this study because it provides a systematic technique for examining and considering various options. It assists decision-makers in gaining access to and selecting from a variety of options (Geoffrion et al., 1972). It strengthens the overall decision-making process by providing a framework for identifying improved qualities across all key measures (Collins et al., 2006). The ABC theory predicts environmental and consumer behavior in a variety of situations. Guagnano et al. (1995) proposed the idea, and it describes how contextual elements might assist in predicting consumer attitudes toward displaying particular behaviors. According to the researchers, simply having a positive mindset is insufficient to legitimize consumer behavior (Goh and Balaji, 2016) in online shopping. It is proposed that the relationship between attitude and behavior is greater when behavior is encouraged by structural conditions at a modest level rather than when it is extended to the point that even individuals with the least favorable attitudes would engage in it (Olander and Thogersen, 2005). This study will evaluate the impact of online customer experience on customer engagement and loyalty, with the help of VCC.

**FIGURE 1:**
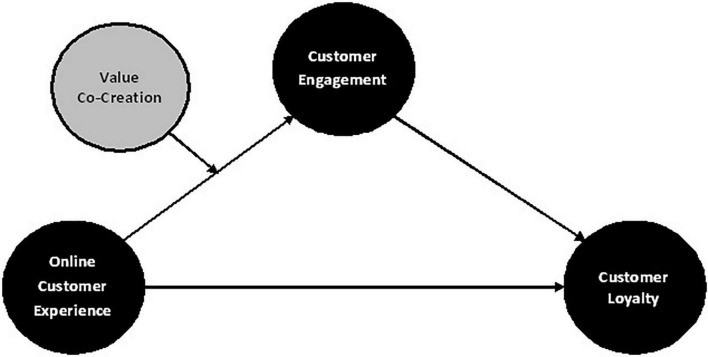
Proposed research model.

According to researchers, online shopping is a low-involvement activity that does not necessitate extensive information gathering. As a result, it falls into the category of habitual purchasing behavior, in which the customer is less involved (Hansen et al., 2004). It was also suggested that when customers shop online, they are likely to use cognitive resources to craft their beliefs about related attributes, leading to an overall attitude toward the behavior (Zaichkowsky, 1985). As a result, the ABC theory has been used to investigate the role of online customer experience in predicting customer loyalty through their engagement in buying groceries online (Guagnano et al., 1995). In addition, VCC is included in the model as a moderator to investigate the customer’s engagement further (see Figure 1). It will assist in determining whether and to what extent customers could be involved, which could help businesses grow and provide a win-win situation for both online retailers and customers.

### Online Customer Experience

Online customer experience is expressed as the impression a customer retains after interacting with products, services, and enterprises and forming a perception based on sensory data (Anshu et al., 2022). It is a critical component of providing customer satisfaction, setting expectations, developing consumers’ faith and gaining confidence, retaining loyal customers, and forming effective ties (Slack and Singh, 2020). Experience is a broad and universal phrase that can be applied to various fields and contexts of study. The topic has received much interest from academics and professionals alike; however, researchers claim that study in this sector is limited and scattered (Bilgihan et al., 2016).

Customer experience, according to Hult et al. (2019), is an internal and subjective reaction that occurs during the process of direct and indirect contact between a customer and an enterprise and includes a variety of aspects of service quality provided by the company, such as advertising, packaging, function, user-friendliness, and product and service reliability. As a result, the availability of a widely accepted study on consumer experiences in retail appears to be restricted (Petermans et al., 2013). According to the literature, online customer experience may be grouped into four categories: product experience, experience with the service procedure, experience with the shopping environment, and experience with staff service (Pei et al., 2020).

### Online Customer Experience and Customer Loyalty

Given that consumer brand loyalty is based on long-term and close contact between a customer and a brand, past research (Mascarenhas et al., 2006; Meyer and Schwager, 2007; Brakus et al., 2009) has found a correlation between customer experience and customer loyalty. According to certain studies, a good customer experience can significantly increase brand loyalty (Biedenbach and Marell, 2010; Morgan-Thomas and Veloutsou, 2013). Lin and Kuo (2013) discovered that recent purchases influence consumers’ loyalty intentions, implying that a pleasant brand customer experience may be the key to strong customer loyalty. Online customer experience is a multidimensional construct, according to Brakus et al. (2009), and all types of customer encounters can influence customer loyalty. Customer loyalty is a crucial determinant of customer experience (Chahal and Dutta, 2015). According to Srivastava and Kaul (2016), it can promote attitudinal and behavioral loyalty to the service provider, while prior research found a strong link between customer experience and loyalty (Klaus and Maklan, 2013). They also believe that customer experience, rather than customer pleasure, predicts a link between service evaluations and consumer behavior and is a stronger predictor of loyalty. Similarly, Sirapracha and Tocquer (2012) claim that it improves customer loyalty, as measured by a lower proclivity to transfer brands.

Customers spend varying amounts of time and effort exchanging information online, offering comments, and participating in decision-making processes (Auh et al., 2007). This research adopts a behavioral approach to capturing customers’ engagement in the service process (Dabholkar, 1990), or, in other words, the extent to which they contribute effort and resources to the online service creation process insofar as they actively participate in consuming and providing value (Nysveen and Pedersen, 2014). Customers who co-produce with a service provider share fresh ideas (Chen et al., 2011), suggestions, and problems with the company, making them feel more fulfilled due to their commitment (Cermak et al., 2011). Customers offer resources to assist the process, according to Ranjan and Read (2016), so co-production is a shared act of satisfaction. It is because customers contribute resources to help with the online process. Their collaboration with a brand increases brand engagement, influencing their brand experience (Nysveen and Pedersen, 2014). As a result, client satisfaction (Flores and Vasquez-Parraga, 2015; Ranjan and Read, 2016) and loyalty (Auh et al., 2007; Nysveen and Pedersen, 2014) are effectively predicted by co-production. Customer–employee online connections can help build customer loyalty (Guenzi and Pelloni, 2004). Even if customers’ expectations are not satisfied, as Lemmink and Mattsson (1998) demonstrate, an employee’s perceived warmth can lead to favorable online views of productivity and loyalty. According to Sivadas and Baker-Prewitt (2000), customers with good online relationships with employees are more likely to return and suggest the company to their friends. Delcourt et al. (2016) emphasized the importance of employee competency and customer happiness and loyalty in particular. Therefore:

*H_1_: Online customer experience positively impacts customer loyalty.*

### Mediating Role of Customer Engagement

Involvement, commitment, passion, enthusiasm, absorption, focused effort, zeal, dedication, and energy are all common connotations of engagement (Schaufeli, 2013). Similarly, the Merriam-Webster dictionary defines engagement as “emotional involvement or commitment” and “getting in gear.” Chebat and Michon (2003) discovered that shoppers’ perceptions of the shopping environment stimulate and alter their emotions in the setting of retail. Online shoppers who are in a better mood before going shopping will have a more favorable impression of the product they see and are more likely to purchase (Puccinelli, 2006). Hence, antecedents of online customer experience have impact over customer engagement. According to Dennis et al. (2010), enhancing the online shopping environment boosts sales and customer spending. There are three dimensions to it. The first is the existing legal framework, which protects customers in online transactions from any type of loss (Li and Zhang, 2002). The second is the third-party recognition system, in which several third-party certification bodies seek to verify that online suppliers are trustworthy (Borchers, 2001). Consumers’ trust in online retailers is favorably correlated with these two criteria. The third criterion is the number of competitors, defined as the number of websites that offer the same service and products (Lee et al., 2000). The fewer the competing vendors, the more likely current merchants will engage in opportunistic behavior to maximize earnings. This raises the consumer’s transaction expenses, lowering their desire to return to a specific online store. This study will focus on environmental effects on customer’s attitudinal and behavioral engagements. The amount to which a website supports efficient and thriving shopping, purchasing, and delivery of items and services is defined as online staff service quality (Zeithaml et al., 2002). Many people believe that the work happiness and engagement of the employees with whom they deal influence client attitudes (Furnham and Milner, 2013). As expected, Tsai and Huang (2002) discovered that staff member friendliness was associated with clients’ pleasant attitudes after a service encounter. Hence, this study elaborate the impact of antecedents of online customer experience on customer engagement in online businesses.

Dabholkar (1996) did an early study on online service quality that looked at how users build expectations about technology-based self-service quality and proposed five primary quality attributes: speed of delivery, simplicity of use, reliability, enjoyment, and control (Rita et al., 2019). Some online firms offer staff service that allows customers to inquire about the product they want to buy in greater detail. Organizations frequently employ digitally synchronous communications such as chat sessions, an online support desk, and social sites (Turel and Connelly, 2013). The impact of online staff service on consumer attitude and behavior is not well documented in the literature. To fill the gap, this study determines the impact of online customer experience on customer attitudinal and behavioral engagement. This research will fill that prerequisite. In terms of capability and efficiency, the online shopping method must fulfill consumer satisfaction as much as feasible, and the efficiency of its operation defines a store’s competitiveness (Artusi et al., 2020). The online shopping procedure’s design and management quality impact the service quality provided and the store’s performance and customer loyalty. Poor business procedure efficiency during online shopping results in a negative consumer experience and discontent (Chen et al., 2020). In terms of the relationship between customer experience and customer satisfaction in the online retail industry, Oliver et al. (1997) stated that incentives and good emotions please customers and contribute to customer happiness. Product experience, according to Zarantonello and Schmitt (2010), can improve consumer satisfaction, product attention, and brand reliability. Hence, these features lead to customer loyalty with the help of customer attitudinal and behavioral engagement in online customer experience. This study will check the mediating role of customer attitudinal and behavioral engagement on the relationship of online customer experience and customer loyalty. According to Šeriæ et al. (2020), it is most likely the outcome of an emotional connection rather than the concept of an emotional link. In contrast, emotion is the result of internal stimulation. The study concludes that product experience is a higher-level analytical and convergent mental experience in nature rather than basic cognitive behavior. It will lead to customer brand appraisal as a critical factor in brand attitude and repeat online purchase behavior. Hence:

*H_2_: Customer engagement mediates the positive relationship between online customer experience and customer loyalty.*

### Moderating Role of Value Co-creation

There is a dearth of empirical evidence of customer value through online customer interaction in a business-to-business scenario. In order to close this gap, the authors looked into and found evidence that consumer participation did result in VCC for customers. Chan et al. (2010) findings further support Mascarenhas et al. (2004) claim that customers’ active online participation in a customer value chain beyond the consumption of an end-product can bring value to customers. Generally, customers today seek engaging experiences that can ultimately take them beyond satisfaction and into the realm of delight. The added value is important because today’s consumers seek satisfaction beyond that provided by an end product or service; instead, they seek engaging experiences that can ultimately take them beyond satisfaction and into the realm of delight (Flores and Vasquez-Parraga, 2015). Individual value is derived through feelings of accomplishment, prestige, personal growth, or enjoyment derived from VCC (Flores and Vasquez-Parraga, 2015). According to previous research, the sensations above are inherently motivating elements for online consumers who are thinking about trying out VCC *via* self-service technology for the first time (Meuter et al., 2005). However, because those sensations are experienced from online participation in the VCC process, they can be legitimately classified as a source of value co-created concerning a customer experience (Dong et al., 2008). Customers in high-participation online environments go so far as to rate their happiness with their performance, according to Matzler et al. (2005), implying that participation might elicit a thoughtful response from customers.

Consumers have recently evolved from passive purchasers of goods and services to active, self-motivated online participants capable of defining and generating value for themselves. As a result, they are no longer a willing and obedient end to a retailer’s marketing efforts but have evolved into a more creative contributor and provider (Anshu et al., 2022). It creates value through the definition, manufacturing, delivery, and usage of products and services by producers and consumers working together. Customers interact online with businesses and exchange ideas during the design, creation, delivery, and final consumption of products, expanding the number of interfaces (Choo and Petrick, 2014). Online businesses can use the concept of VCC as one of their retention tactics. According to studies, online co-creation amplifies the impact of experiences, improving perceived value, positive attitude (Meng and Cui, 2020), purchasing intention, and behavior (Pee, 2016). According to research, VCC has a moderating effect on the outcomes of experiences (Meng and Cui, 2020). Customers return to retailers who appreciate their ideas, which has resulted in online customer traffic being directed to companies where their ideas are sought, appraised, and included, resulting in VCC (Pathak et al., 2017). However, depending on the type of service, the level of VCC might range from minimal to high. Despite its acceptance, actual research on the results of co-creation is sparse (Carbonell et al., 2009). This research offers to investigate the interactions supported by VCC (the operational state), in which it functions as a moderator to further investigate the customer’s engagement purpose. This will aid in determining if customers can participate in VCC in online stores and, if so, to what extent this may assist businesses in growing and providing a win-win situation for both (Anshu et al., 2022). As a result, the researcher wanted to see if online stores might take advantage of this concept and use it to grow their consumer base and bring loyalty to the relationships.

*H_3_: Value co-creation moderates the relationship between online customer experience and customer engagement, such that the relationship is stronger when value co-creation is higher.*

## Materials and Methods

### Pilot Study

A pilot study was done who were using internet purchasing participants. A total of fifty young adults took part in the pilot trial. They were invited to share their online store and brand experiences. Customers stated that they shop online *via* online application platforms such as Facebook and Instagram. They also discussed the brands that they purchased from internet retailers. They bought outfits, footwear, jewelry, bracelets, cellphones, and other fashion products. The researchers used the pilot study results to determine what consumers want and how they buy from online retailers. As a result, it provided context for this study and mapped out the research process.

### Sample and Technique

A cross-sectional survey acquired information through a structured online questionnaire from young adult customers purchasing things online in Pakistan. The reason for selecting youthful customers is that they are more likely to be loyal to online retailers in the long term and are more eager to try out new things (Kumar, 2021). The online poll was distributed over several online platforms, and only those performing online shopping were eligible to participate. The information was gathered between January 15th and February 14th, 2022. There were 196 responses out of which 189 valid responses were used for further investigation. The demographic data are presented in Table 1. The statistical programs SPSS and Smart-PLS were employed to analyze the data and draw inferences. Participants were chosen based on their age limit and online buying experience using the judgmental sampling (non-probability sampling) method. The age range was set at 16–35 years old, and they had to have done some internet shopping at least once. This strategy was also used as the first question in a questionnaire. Participants who did not match the criteria were not endorsed to continue the survey. The data was collected from the target sample in this manner. The demographic results are shown in Table 1.

**TABLE 1:** Participants’ demographic details.

Characteristics	Participants (*N* = 189)	
	Frequency	Percentage
*Gender*		
Male	137	72.5
Female	52	27.5
*Age*		
16–20 year	69	36.5
21–25 year	98	51.9
26–30 year	10	5.3
31–35 year	12	6.3
*Educational qualification*		
Doctoral	9	4.8
Masters	25	13.2
Graduation	106	56.1
Intermediate	49	25.9
*Occupation*		
Student	18	11.6
Business	90	58.1
Service	37	23.9
Self-employed	10	6.5
Housewife	10	6.5
Other	10	6.5
*Monthly household income (US$ 1* = *PKR 186.5)*		
Less than Rs. 25,000 (<$134.5)	8	5.2
Rs. 25,000–49,999 (up to $268.09)	24	15.5
Rs. 50,000–74,999 (up to $402.14)	70	45.2
Rs. 75,000–99,999 (up to $536.18)	53	34.2
Rs. 100,000 or More (> $536.18)	10	6.5
*Time duration since[2pt] buying products online*		
Less than 1 month	56	36.1
1–6 months	44	28.4
6–12 months	23	14.8
More than 1 year	32	20.6
*Online products purchase frequency*		
Daily	56	36.1
Once a week	44	28.4
Fortnightly	23	14.8
Monthly	32	20.6
Rarely	32	20.6
*Value of the online shopping (Rupees per purchase)*		
Rs. 1,000 or less (<$5.36)	56	36.1
Rs. 1,001–2,000 (up to $10.72)	44	28.4
Rs. 2,001–3,000 (up to $16.09)	23	14.8
Rs.3,001–4,000 (up to $21.45)	32	20.6
Rs. 4,001–5,000 (up to $26.81)	32	20.6
Rs. 5,001 or More (>$26.81)	32	20.6

### Measures

This study used validated measures to collect data, utilizing a 7-point Likert scale ranging from 1 (strongly disagree) to 7 (strongly agree). Furthermore, the scaled items of variables were given hereunder.

#### Online Customer Experience

This study used four dimensions of online customer experience named product experience, shopping environment, staff service experience, and shopping procedure. All four dimensions of online customer experience and their scale were adapted from Parasuraman et al. (1988). Product experience (PE, α = 0.74) contains five items; such an item is “This online store has a variety of categories and colors.” The shopping environment (SE, α = 0.79) comprises three items; one of the items is “This online store shows neat and attractive web design.” Staff service experience (SSE, α = 0.81) has three items: “The staff of this online store shows frequent communication with the customer and good service attitude.” At last, the shopping procedure (SP, α = 0.77) has three items: “This online store makes sure the availability of pictures and reviews at all times.” All four measures of online customer experience indicated good reliability in this study (PE: CR = 0.90, α = 0.87; SE: CR = 0.89, α = 0.85; SSE: CR = 0.94, α = 0.92 and SP: CR = 0.89, α = 0.85).

#### Customer Loyalty

Brakus et al. (2009) adapted the customer loyalty scale, containing three items (α = 0.63). One of the items is “I intend to stay on as a customer of this online store for the next 5 years.” The scale (CR = 0.95, α = 0.93) showed good reliability in this study.

#### Customer Engagement

This scale contains two dimensions; attitudinal engagement and behavioral engagement. These two dimensions have been adapted from Vivek et al. (2014), in which attitudinal engagement (AE, α = 0.74) has six items (“Anything related to this online store grabs my attention”) and behavioral engagement (BE, α = 0.74) has four items (“I provide this online store some useful suggestions to improve services”). Both measures of customer engagement indicated good reliability (AE: CR = 0.90, α = 0.87; BE: CR = 0.89, α = 0.85).

#### Value Co-creation

The scale of VCC is adapted from Prahalad and Ramaswamy (2004) in the form of six items (α = 0.63), for example, “I am actively involved when this online store develops new solutions for me.” The measure of VCC exhibited good reliability (CR = 0.95, α = 0.93). This study used four dimensions of online customer experience named product experience, shopping environment, staff service experience, and shopping procedure. All four dimensions of online customer experience and their scale were adapted from Parasuraman et al. (1988). Product experience (PE, α = 0.74) contains five items: “This online store has a variety of categories and colors.” The shopping environment (SE, α = 0.79) comprises three items; one of the items is “This online store shows neat and attractive web design.” Staff service experience (SSE, α = 0.81) has three items: “The staff of this online store shows frequent communication with the customer and good service attitude.” At last, the shopping procedure (SP, α = 0.77) has three items: “This online store makes sure the availability of pictures and reviews at all times.” All four measures of online customer experience indicated good reliability in this study (PE: CR = 0.90, α = 0.87; SE: CR = 0.89, α = 0.85; SSE: CR = 0.94, α = 0.92 and SP: CR = 0.89, α = 0.85).

## Results

### Measurement Model

**FIGURE 2:**
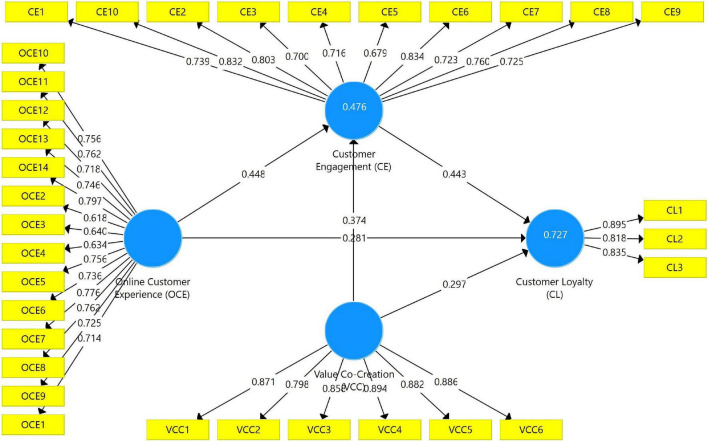
Measurement model.

The measurement model used in this study showed a high construct validity and reliability level. Except for one item for online customer experience (OCE = 0.566), almost all factor loadings surpassed the benchmark of 0.700 in Figure 2. Cronbach’s Alpha (>0.700), composite reliability (>0.700), and average variance extracted (AVE) values over 0.500 fulfilled the minimal standards, as shown in Table 2. All constructions’ composite reliabilities were likewise higher than their respective AVEs. The study also looked at discriminant validity, which requires that the square root of all AVEs be greater than the correlations between constructs (Fornell and Larcker, 1981). Table 3 reveals that square roots of AVEs are larger than inter-construct correlations, indicating that discriminant validity has been established. As a result, the structural model’s hypothesis testing assumed that the measurement model was sufficiently trustworthy and valid.

**TABLE 2:** Construct reliability and validity.

	α	CR	AVE
Online customer experience	0.930	0.940	0.527
Customer engagement	0.914	0.929	0.567
Value co-creation	0.933	0.947	0.749
Customer loyalty	0.808	0.886	0.722

*α, Cronbach’s Alpha; CR, Composite Reliability; AVE, Average Variance Extracted.*

**TABLE 3:** Correlations and discriminant validity.

	Mean	SD	1	2	3	4
Online customer experience	5.504	1.067	**0.726**			
Customer engagement	5.046	1.163	0.599**	**0.753**		
Value co-creation	4.980	1.371	0.405**	0.555**	**0.865**	
Customer loyalty	5.273	1.176	0.667**	0.737**	0.657**	**0.850**

n = 189, **p < 0.01, SD, Standard Deviation; Bold diagonal values are √AVE.

### Descriptive and Correlational Analyses

The mean, standard deviations, and correlation coefficients for the research variables are shown in Table 3. As expected, online customer experience indicated significant positive correlations with customer engagement (β = 0.599, *p* < 0.01) and loyalty (β = 0.667, *p* < 0.01). VCC reflected a significant positive correlation with customer engagement (β = 0.555, *p* < 0.01) and loyalty (β = 0.657, *p* < 0.01). In this investigation, these correlations revealed the possibility of forecasting theoretical connections without the danger of multicollinearity.

### Structural Model and Hypothesis Testing

**FIGURE 3:**
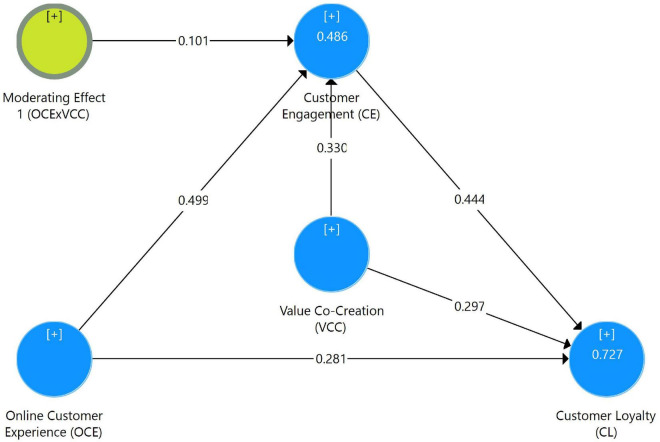
Estimated path model.

The path analysis in this study was done using a partial least square (PLS) method. The direct, indirect, and total effects were studied to evaluate the proposed hypotheses, as indicated in Table 4. To begin, the study looked at the direct and cumulative effects of online customer experience (OCE) on customer loyalty (CL) and customer engagement (CE). Second, the direct and total effects of OCE on CE and the effects of CE on CL were investigated. Finally, the four-step approach (Baron and Kenny, 1986) was used to observe the mediation impact of CE on the link between OCE and CL. Finally, the analysis examined the moderating effects of VCC on CE and CL, respectively. The complete moderated-mediation model and the estimated PLS path model (Figure 3) show that OCE (β = 0.499), VCC (β = 0.330), and their interaction term (OCE × VCC; β = 0.101) explained a 48.6% variance in CE (*R*^2^ = 0.486). The overall model explained a 72.7% variance in customer loyalty (*R*^2^ = 0.727).

**TABLE 4:** Path analysis.

Path	Effect (*t*-value)	Hypotheses	Outcome
OCE → CL	0.502* (6.834)	H_1_	Supported
OCE → CE → CL	0.221** (4.281)	H_2_	Supported
VCC → CE	0.330** (4.362)	−	−
VCC → CE → BL	0.147** (2.715)	−	−
Moderating effect 1: OCExVCC→ CE	0.101* (1.626)	H_3_	Supported

**p < 0.01, *p < 0.05; CE, Customer Engagement; CL, Customer Loyalty; OCE, Online Customer Experience; VCC, Value Co-Creation.

The OCE has a substantial positive relationship with CL. The total (β = 0.502, *t* = 46.834, *p* < 0.01) of OCE on CL was positive and significant, validating hypothesis 1 (see Table 4). According to Hypothesis 2, CE mediates the link between OCE and CL. Given that hypothesis 2 satisfy the mediation requirements (Baron and Kenny, 1986), the stated path analysis (see Table 4) indicated that OCE has an indirect influence on CL (β = 0.221, *t* = 4.281, *p* < 0.01) through CE. As a result, hypothesis 2 was found to be true. VCC positively moderates the associations between OCE and CE, according to hypothesis 3. The results of the path analysis revealed that VCC made a significant contribution to CE (β = 0.330, *t* = 4.362, *p* < 0.01) and its moderating effect (OCE × VCC) on CE (β = 0.101, *t* = 1.626, *p* < 0.05) was positive and significant, supporting and implying that the link between OCE and CE grows stronger at greater levels of VCC.

## Discussion

According to the findings, customer engagement, i.e., attitudinal and behavioral engagement, are significantly correlated with the shopping environment, shopping procedure, product experience, and staff service experience. According to the study, customer engagement has a significant positive relationship with customer loyalty. Hypotheses H_1_, H_2_, and H_3_ were therefore accepted.

Hypothesis 1: By highlighting the relationship in an online setting in an e-commerce environment. Based on these findings, it can be concluded that an online customer experience, which is a one-time event, directly impacts a long-term phenomenon like customer loyalty. However, because a better online flow state leads to a better overall brand experience, it aids in the development of stronger client loyalty (Ertemel et al., 2021). The findings show that investing in a flawless online experience that puts customers in the flow keeps them amused and improves brand loyalty over experience.

Hypothesis 2: In the long run, the online customer experience improves consumer involvement with the company (Brodie et al., 2013; Wirtz et al., 2013). Innovative digital channels and customer touchpoints, such as social media and mobile devices, for example, allow brand marketers to engage customers in new ways (Chan, 2012; Sashi, 2012), while customers have more opportunities to interact with their favorite brands and stay active in online brand communities (Brodie et al., 2013). In the context of online gaming, Luo et al. (2010) empirically evaluated and found favorable results that improving online customer experience promoted customer loyalty, notably repeat purchases and WOM. Wirtz et al. (2013) summarized the existing research on online consumer engagement. They argued that online brand communities give new avenues for customers to connect with the brand while also increasing customer involvement. Attitudinal engagement improves positive attitudes about a company or speeds up the shift from attitudinal to behavioral loyalty (So et al., 2016; Harrigan et al., 2017). According to studies, increased brand engagement in self-concept as an attitudinal component of consumer engagement leads to higher customer loyalty (Sprott et al., 2009). Hence, the mediation of customer engagement is confirmed in this study regarding online customer experience and loyalty.

Hypothesis 3: The client is no longer merely the end recipient of products and services in the new reality of internet commerce. They have turned into players in their value generation. The product in VCC is a development in which the consumer becomes enthralled and participates in the process (Auh et al., 2007). Improved levels of co-creation have been linked to increased customer engagement and the desire to co-create in the future (Frasquet-deltoro and Lorenzo-romero, 2019), resulting in a rise in the number of interactions (Choo and Petrick, 2014) and loyalty. Companies may use the notion of VCC as one of their retention tactics in the form of loyalty. Studies show that customer experience and the VCC process are inextricably linked; as a result, online co-creation amplifies the impact of experiences, enhancing perceived value and positive attitude (Meng and Cui, 2020), and that positive attitude leads to customer loyalty (Barari et al., 2020). The investigation also revealed that VCC has a strong moderating influence on all interactions. Except for the Delivery experience, this effect was stronger at lower levels of VCC and declined as the amount of co-creation grew.

### Theoretical Implications

This study created and experimentally tested a complete OCE-ABC model to examine the impact of factors that precede OCE on consumer behaviors and loyalty in online buying. The study established an ABC and MAUT conceptual model based on current literature, framework, and theories. Although numerous researches have been undertaken in internet enterprises, very few studies are accessible in online purchasing. Also offered is an all-inclusive moderation model that considers the convergence of e-commerce, customer experience, customer engagement, and new elements supplied by the internet, such as networking effects and VCC. This has not been mentioned in the current internet shopping literature.

Second, past research has revealed that the shopping process, product experience, shopping environment, and staff services are all essential factors in determining customer loyalty; however, none of these factors positively or substantially impacted the consumer’s attitude in this study. This was addressed in the study’s discussion section. These aspects should be considered, and any obstacles should be removed to motivate people to buy items online. It was also shown that recovery has emerged as a critical component in consumer behavior and online retail loyalty.

The inclusion of VCC as a moderator in the model then helped to understand better consumers’ intent and expectations in an online shopping scenario. Except for the delivery experience, the numerous interactions revealed that, in the current scenario, co-creation has a minor impact in shaping the various relationships between the antecedents and the consumers’ attitudes and behavior toward online shopping. This revealed that customers who buy things online are more engaged in co-creating value with businesses to improve their shopping experience.

Overall, the findings are exciting and contribute significantly to the online customer experience literature in e-commerce. The study may benefit academics who want to learn more about the factors that influence OCE’s attitude and behavior and the impact of VCC with enabled and empowered consumers in the ever-changing and overwhelming world of e-commerce. In light of the ABC theory and MAUT in the current environment of online experiential, the study contributes to the conceptual model for OCE and customer loyalty in online shopping.

Existing research has looked at characteristics that impact customer loyalty in physical stores, but it has not looked at the link between customer experience and loyalty in online buying. Existing research also fails to recognize the uniqueness of the circumstance in which online store business mixes and develops. The current study used empirical research and included the component of VCC as a moderating variable to investigate changes in characteristics that impact consumer loyalty in online purchasing, overcoming the shortcomings of earlier studies to some extent. Second, the research looked at the link between customer experience and loyalty in the complex world of retail, which has seen the merger of online and brick-and-mortar stores. It systematically investigated the impact of the consuming process on customer loyalty in various online stores from the perspective of online customer experience, contributing to the theory of the experience mechanism of how customer loyalty takes shape. This provides for more in-depth, methodical, and complete customer loyalty research. As a result, the theoretical contribution combines and expands the link between online customer experience, loyalty, and long-term competitive advantage. The introduction of new technologies, such as internet technology, has increased the rivalry in the retail business. Particularly in technology-driven sharing economies, the need for joint and collaborative consumer activities is a mounting trend (Graessley et al., 2019). This has led to a widespread effect on consumers’ online shopping trust and their decision-making regarding online business activities. As a result, the link between online customer experience, loyalty, and long-term competitive advantage has grown stronger.

The model’s validation showed numerous vital implications for the literature on customer engagement behavior, mainly organic and encouraging engagement routes. Through perceived online value, functional and experiential activities drive customer engagement directly and indirectly. Previous research on firm-initiated engagement (Ryu and Feick, 2007; Tafesse, 2016; Wirtz et al., 2019) has only looked at the direct influence of firm-initiated activities on engagement. However, the current study shows that the impact of functional and experiential activities is not restricted to direct consequences. They are linked to the organic pathway *via* perceived online worth. Furthermore, experiential activities have a significantly more direct influence than operational initiatives (Hilken et al., 2021).

The link between the attitudinal and behavioral components of customer involvement is also explored in this study. Although the literature supports the motivational driver of engagement behavior (van Doorn et al., 2010; Lemon and Verhoef, 2016), the importance of behavioral engagement for firm performance encourages researchers to concentrate on this component (Kumar and Pansari, 2015). While the results acknowledge the significance of behavioral engagement and believe that attitudinal engagement is as essential in creating engagement. The correlation between commitment and experimental efforts with attitudinal engagement is significantly more significant in both organic and promoted engagement than the link between commitment and active–behavioral engagement. Attitudinal components appear to be more important than behavioral components in establishing engagement (Barari et al., 2020). Moreover, other important constructs like relationship quality and brand love should also be included in future research.

Furthermore, no study on the interaction between customer engagement components has been done. Attitudinal engagement, like staff engagement (Saks, 2006; Shuck and Wollard, 2010), is a necessary precondition for behavioral engagement. These findings emphasize the importance of attitudinal involvement in establishing customer engagement.

The findings show that the connections between attitudinal and behavioral engagement and their consequences, such as customer loyalty and firm performance, are confined to attitudinal engagement–loyalty and behavioral engagement–firm performance. According to the cognitive-affective-behavioral hierarchy, attitudinal involvement was a rational predictor of customer loyalty (Oliver, 1999). Similarly, the findings show that behavioral engagement can directly influence firm performance (Kumar and Pansari, 2015; Beckers et al., 2017) and that attitudinal engagement has an indirect impact on performance through behavioral engagement. These findings help to clarify the connection between engagement and its outcomes. Speculative attitudes and behaviors, such as referring to a new customer or making a social media comment, are required for customer interaction.

### Managerial Implications

This research also has some practical implications for online businesses that want to pay attention to the essential factors influencing the customer experience. Customer experience is a multifaceted component, and the impact each of its antecedents has on customers’ attitudes and online retailers must recognize loyalty. They must also understand the value of VCC in the changing environment and its influence on the total online customer experience and its consequences.

The findings revealed that the shopping procedure significantly impacted customers’ attitudes and behavioral intentions regarding online shopping. Customers are concerned about finding out where their things are and when they will be delivered. They save time and avoid the stress of standing in lengthy lines thanks to the product’s ease and doorstep delivery. They want to be a part of the co-creation process to create value through an easy purchasing process. This is because the customer has already committed time, energy, and, in some instances, money in purchasing the items and so attempts to become more active in the co-creation process to guarantee that delivery is efficient. As a result, it is proposed that businesses aim to include customers in developing efficient shopping procedures.

Another critical aspect of merchants’ loyalty was efficient staff service. Customer complaints may be turned into satisfaction and converted into loyal customers (Miller et al., 2000) with the support of efficient and effective staff service. Weak recovery may disappoint consumers for the second time, resulting in lousy WOM and customers moving to rivals (Lewis and Mccann, 2004). Another criterion that did not directly impact online shopping loyalty was the antecedent product experience. However, in the presence of co-creation as a moderator, these characteristics affected consumers’ desire to repurchase considerably, albeit at a lesser degree of involvement.

Customers’ expectations should be used by online shops to co-creating value for them and gain their loyalty. Circumstances directly influenced customers’ attitudes before OCE, influencing their online customer loyalty. However, at a lower degree of co-creation than at a greater level of co-creation, co-creation served as a moderator for most of the antecedents. This might be because customers regard purchasing things online as a low-involvement activity, and investing more time and energy in them is not seen as providing a value proposition. As a result, online shops must ensure that clients receive the most valuable experience possible. The study supports the ABC theory, which states that when structural factors enable behavior at a moderate level, the association between attitude and behavior is higher, but not to the point where even people with the least favorable attitudes would execute it (Olander and Thogersen, 2005). As a result, regularly including consumers in co-creating an excellent customer experience may be a helpful approach for online merchants to improve online customers’ attitudes and repurchase intent in online buying. Customers may be influenced by the outcomes in the future, making them more inclined to purchase online.

Online website customers are increasingly expecting to be engaged in extraordinary experiences that allow them to experience a sense of time and space distortion without thinking about superfluous details. This is especially true in today’s attention economy-driven, highly fragmented, and hectic daily lives. In such an environment, customers are inundated with multiple impulses, making it impossible to devote their limited and essential time and attention to any of them. As a result, online shopping may be regarded as a break from the daily grind, and as a result, today’s customers place a higher value on it. The findings show that investing in a smooth online experience that puts clients in the flow keeps people amused and improves brand loyalty and customer satisfaction in the long run.

Gummerus et al. (2012) and Wirtz et al. (2013) found that the online brand community delivers various unique benefits that improve customers’ experiences with the firm’s products and services. The marketing manager can directly use functional and experiential activities to affect customer engagement in the promoted strategy directly. Experiential activities are particularly successful in generating attitudinal engagement, whereas functional initiatives have a relatively minimal impact on behavioral engagement. Suppose marketing managers assess their marketed strategy based on short-term impact. In that case, they should invest more in experiential initiatives in the form of a game or event, especially on social media, to indirectly affect consumer engagement behavior and attitudinal engagement.

Furthermore, perceived value influences consumer engagement through functional and experiential activities through organic pathways. As a result, marketing managers might mix organic and promoted engagement techniques to target current consumers with a long-standing connection. This strategy might improve customer engagement attitudes and behaviors by creating synergy between the two engagement tactics.

The following areas of a company’s processes should be improved. To boost consumer experience and loyalty, merchants must first implement online store retail. It may assist merchants in gaining a better and faster knowledge of the consumer experience and long-term competitive advantage goals. Retailers should: (1) diversify their promotional methods, attracting customers through multiple channels and building strong relationships with them through online marketing on mobile devices; (2) generate traffic through social media and promotional coupons for customers and followers; and (3) use affiliate marketing to penetrate target customer groups.

Second, shops must exercise sales management and recognize the importance of internet buying. They should establish a scientific and practical sales management system, consider the impact of different situations and genders on the relationship between OCE and customer loyalty, provide the best shopping experience for customers, build a good brand image, and launch customer-centric marketing campaigns in order to achieve a sustainable competitive differentiation. Finally, businesses should link their online and physical store channels to give customers the ideal buying experience: (1) They should employ digital technology to create two-way online store commerce interconnectivity; (2) they should link the coupling channel of online store commerce and create a comprehensive system network; (3) They should rely on system synergy and data analysis capabilities to help them optimize OCE and effectively maintain long-term customer loyalty.

### Limitations and Future Research Directions

This work has certain limitations, and researchers in the future may revisit it. The population of online shoppers in various geographic locations and other nations may not be represented in the sample analyzed. As a result, the study needs to be duplicated on a bigger sample size in diverse and online consumers in other countries to generalize the findings. The study focused on cost and time restrictions and mainly was a cross-sectional, one-time survey study. On the other hand, long-term longitudinal research would make a more significant contribution to knowledge. This study focused on business-to-consumer (B2C) online commerce and may not apply to other online business categories such as B2B or others.

Further research on these aspects and other new elements in the field of OCE might be conducted to examine various behavioral intents and results. The previous study has mainly focused on customer engagement as a positive term, although engagement behavior contains both good and negative aspects. Future research may consider other dimensions of online customer experience as well. Such dimensions may be applied on this model, for example convenience, privacy and security, network effect and recovery as antecedents of online customer experience.

Furthermore, this model suffers from customer-related aspects since past research has concentrated on firm-related antecedents and consequences of engagement. Consumer relationship creation and customer reaction to advertised engagement activities, for example, will be influenced by psychological variances among customers. Similarly, because it lacks customer-related outcomes, the result simply reflects the firm’s advantages of customer involvement.

## Conclusion

Due to the rising usage of online media, e-commerce adoption rates surged even more due to the COVID-19 epidemic. Providing flawless experiences and building long-lasting, lucrative connections with clients have become more vital than ever during these trying times for e-businesses. The findings of this study show that online purchasing, as regarded by consumer trust among young adults in Pakistan, has an indirect impact on their loyalty to e-commerce companies through customer experience. Online shopping aims to provide a completely immersive and engaging experience with a brand’s website. Naturally, this event is predicted to occur soon.

As a result, theoretically, it is unlikely to impact long-term phenomena such as consumer loyalty. Even though online shopping has been investigated from several perspectives, the number of studies evaluating the impact of online shopping on the entire brand experience and consumer loyalty is limited in the current literature. However, only the customer experience-loyalty links were examined in that study. Using Smart-PLS, this study attempted to contribute to the existing research by introducing the customer loyalty dimension and assessing the direct and indirect effects of all of those constructs’ phenomena combined.

The findings suggest that in an e-commerce scenario, encouraging a flow state should not be considered a nice-to-have feature. However, doing so aids e-commerce companies in achieving their long-term goals. When it comes to customer loyalty, the instant, a client accesses an online platform for a product, the length of time he or she spends there, and the pleasure obtained are essential factors. Customized and individualized products and influencing aesthetic designs on online platforms produce a more crucial customer experience than the purchase experience, enabling and making the shopping process joyful. In conclusion, websites that keep the client engaged will be favored.

This study attempts to supplement previous research on customer attitudes and behaviors in online buying and loyalty. The study used the Multi-Attribute-Utility theory and the ABC theory to investigate retailing on an online platform, focusing on VCC. Customers and retailers collaborate to develop products and services. The OCE as antecedents of customers’ engagement and loyalty towards online buying has been investigated in this study. This research also investigated and found a positive moderating effect of VCC on the relationship between OCE and consumer engagement with online shopping.

## Data Availability Statement

The raw data supporting the conclusions of this article will be made available by the authors, without undue reservation.

## Ethics Statement

Ethical review and approval was not required for the study on human participants in accordance with the local legislation and institutional requirements. The patients/participants provided their written informed consent to participate in this study.

## Author Contributions

All authors listed have made a substantial, direct, and intellectual contribution to the work, and approved it for publication.

## Conflict of Interest

The authors declare that the research was conducted in the absence of any commercial or financial relationships that could be construed as a potential conflict of interest.

## Publisher’s Note

All claims expressed in this article are solely those of the authors and do not necessarily represent those of their affiliated organizations, or those of the publisher, the editors and the reviewers. Any product that may be evaluated in this article, or claim that may be made by its manufacturer, is not guaranteed or endorsed by the publisher.
